# Analysis of dose comparison techniques for patient‐specific quality assurance in radiation therapy

**DOI:** 10.1002/acm2.12726

**Published:** 2019-10-15

**Authors:** Liting Yu, Timothy L. S. Tang, Naasiha Cassim, Alexander Livingstone, Darren Cassidy, Tanya Kairn, Scott B. Crowe

**Affiliations:** ^1^ Royal Brisbane and Women's Hospital Herston Qld. Australia; ^2^ Queensland University of Technology Brisbane Qld. Australia; ^3^ Beacon International Specialist Centre Petaling Jaya Malaysia

**Keywords:** dose comparison technique, gamma evaluation, PSQA, radiation therapy

## Abstract

**Purpose:**

Gamma evaluation is the most commonly used technique for comparison of dose distributions for patient‐specific pretreatment quality assurance in radiation therapy. Alternative dose comparison techniques have been developed but not widely implemented. This study aimed to compare and evaluate the performance of several previously published alternatives to the gamma evaluation technique, by systematically evaluating a large number of patient‐specific quality assurance results.

**Methods:**

The agreement indices (or pass rates) for global and local gamma evaluation, maximum allowed dose difference (MADD) and divide and conquer (D&C) techniques were calculated using a selection of acceptance criteria for 429 patient‐specific pretreatment quality assurance measurements. Regression analysis was used to quantify the similarity of behavior of each technique, to determine whether possible variations in sensitivity might be present.

**Results:**

The results demonstrated that the behavior of D&C gamma analysis and MADD box analysis differs from any other dose comparison techniques, whereas MADD gamma analysis exhibits similar performance to the standard global gamma analysis. Local gamma analysis had the least variation in behavior with criteria selection. Agreement indices calculated for 2%/2 mm and 2%/3 mm, and 3%/2 mm and 3%/3 mm were correlated for most comparison techniques.

**Conclusion:**

Radiation oncology treatment centers looking to compare between different dose comparison techniques, criteria or lower dose thresholds may apply the results of this study to estimate the expected change in calculated agreement indices and possible variation in sensitivity to delivery dose errors.

## 1 INTRODUCTION

Patient‐specific pretreatment quality assurance (PSQA) tests are routinely performed for the verification of modulated radiation therapy treatment delivery. Modulated treatment modalities, including intensity modulated radiation therapy (IMRT), volumetric modulated arc therapy (VMAT) and helical tomotherapy (HT), achieve highly conformal dose distributions by optimization of dose rates and multileaf collimator (MLC), gantry, couch and collimator movements.^1^ The complexity of MLC movement and the treatment planning system (TPS) dose calculation limitations (accuracy of beam modeling and the algorithm itself) are two of the multiple factors that can introduce disagreement between planned and delivered dose, which impact the accuracy of treatment delivery.

The most common form of PSQA involves the comparison of TPS dose calculations with 2D or 3D dose measurements.^2^, ^3^, ^4^, ^5^ The gamma evaluation method (also known as gamma analysis, or gamma index analysis), developed by Low et al.^6^, ^7^ is widely used to compare such measurements.^5^ This technique compares an evaluated (usually measured) dose distribution with a reference (usually calculated) dose distribution in a quantitative manner by calculating the gamma value of each point, which is the minimum Euclidean distance in the dose‐spatial domain. The agreement between evaluated and reference dose distributions is calculated using two acceptance criteria: dose difference, ∆D, in %; and distance‐to‐agreement, DTA, in mm. Gamma analysis produces gamma index values assigned to each individual point, for which gamma index values ≤1 indicate passed or otherwise failed. The percentage of passing points in the gamma distribution is referred to as gamma pass rate (or %GP). %GP can be used by the users to establish or apply action levels. Surveys of PSQA practices have reported that ∆D and DTA criteria of 3%/3 mm are the most frequently used.^2^, ^3^, ^4^

Global gamma evaluation normalizes the percent differences for every point to a globally used single value, usually the maximum planned dose; whilst local gamma evaluation normalizes the percent differences for every point to the expected dose at each point. Thus, the %GP calculated by global gamma will always be higher than or equal to local gamma, where the same criteria and lower dose threshold are used.^8^ The use of global gamma evaluation for PSQA using ∆D and DTA criteria of 3%/3 mm has been questioned due to reported poor sensitivity and specificity to delivery errors^9^, ^10^, ^11^, ^12^, ^13^, ^14^, ^15^ and clinically relevant patient dose errors,^11^, ^16^, ^17^ and a lack of clinical intuitiveness.^17^, ^18^ Some authors have proposed DVH‐based QA metrics as a response to these criticisms.^17^ A number of studies have proposed or evaluated alternative dose comparison techniques, or assessed variation in behavior of gamma evaluation for both local and global gamma evaluations with different criteria and lower dose thresholds (LDT).^19^, ^20^

Jiang et al.^18^ proposed the maximum allowed dose difference (MADD) technique, in which a distance‐to‐agreement criterion is converted to a dose difference by multiplying the dose gradient at the point of interest. This DTA‐equivalent dose criteria is combined with ∆D to determine MADD (by summation for box calculation MADD*_b_*, or summation in quadrature for gamma calculation MADD*_γ_*). The difference between dose distributions at the point of interest can then be normalized by local MADD (as a “normalised dose difference”), providing an index in which values ≤1 indicate agreement. The advantage of this method over the gamma method is that it is not only accurate and simple but also clinically intuitive and insensitive to dose grid resolution.

Stojadinovic et al.^14^ proposed the divide and conquer (D&C) gamma method, in which the determination of agreement between dose distributions is dependent on the dose region: a high dose (HD) region within the 90% isodose, a high gradient (HG) region between the 90% and 50% isodoses, a medium dose (MD) region between the 50% and 20% isodoses, and a low dose (LD) region between 20% to 10% isodoses. Significant differences in behavior were reported, when D&C results were compared to conventional gamma evaluation, for a dataset containing 50 PSQA measurements.^14^ This method has challenged the adequacy of conventional IMRT QA program. The authors concluded that a better paradigm would be needed to standardize IMRT QA practices. The advantage of the D&C method over the gamma method is that by analyzing four distinct regions separately, more reasonable characterization of the agreement between calculations and measurements can be performed without combining regions of high and low dose gradients.

Some studies have analyzed the effect of induced error to the PSQA results using gamma method.^21^, ^22^ Other studies have characterized the effect of ∆D and DTA criteria selection on gamma agreement indices. Crowe et al.^23^ reported that global gamma agreement indices calculated using 2%/3 mm, 3%/2 mm, and 3%/3 mm were correlated with each other, suggesting that these criteria would produce similar PSQA results (or similar sensitivity and specificity), if action thresholds were adjusted accordingly. Recommendations were provided for radiation oncology treatment centers intending to transition to tighter global gamma evaluation ∆D and DTA criteria.^23^

However, although optimal gamma parameters and performance of alternative metrics were tested in the literature^19^, ^20^, ^24^, few studies^8^ have provided suggestions to radiation oncology treatment centers looking to compare between these alternative dose comparison techniques, between different ∆D and DTA criteria pairs (for local gamma evaluation techniques) or LDT. This study evaluated the D&C, MADD, local and global gamma dose comparison techniques across a variety of agreement criteria and LDT, for 429 existing PSQA measurements obtained using ArcCheck helical diode array (Sun Nuclear Corporation, Melbourne, FL), including 57 beams that failed departmental quality assurance tests, for both HT and VMAT treatments. The percentage of failed plans included in this study is approximately the same as in actual clinical occurrence. A “failed” plan is defined simply by the numerical pass rate and our institutional acceptance criteria: most “failed” plans in this study were considered clinically acceptable when reviewed by a Radiation Oncologist.

## 2 MATERIALS AND METHODS

The global gamma analysis, local gamma analysis, D&C and MADD methods were implemented in Matlab version R2015b (MathWorks, Massachusetts, USA), per original descriptions by Low et al.,^6^, ^7^ Stojadinovic et al.^14^ and Jiang et al.,^18^ respectively. The implementation of the gamma analysis and MADD methods was validated using data described by Low and Dempsey.^7^, ^18^

The in‐house software was designed to iterate through routinely prepared PSQA directories, containing TPS calculated dose distributions, converted to the “.snc” format using Sun Nuclear SNC Patient Software version 6.2.2 (Sun Nuclear Corporation, Melbourne, USA), and ArcCheck measured dose distributions, in “.txt” format. The lower resolution (1 cm) ArcCheck measured dose distribution was compared with the higher resolution (1 mm). TPS calculated dose distribution without interpolation. The VMAT arcs were measured in absolute dose whereas the HT beams were measured in relative dose. Normalization was performed at dose maximum.

An overview of the QA measurements selected for this study is shown in Table 1. This cohort included both measurements that passed departmental PSQA and those that failed. The passed measurements are those that produce global %GPs ≥95% at 2%/2 mm and LDT of 5% with Measurement Uncertainty Corrections turned on in the SNC Patient Software. The Measurement Uncertainty Correction is a default option in the SNC Patient Software intended to compensate for presumed sources of measurement uncertainty that potentially decrease the calculated pass rate. It typically adds about 1%‐2% to the user‐defined acceptance criterion of percentage difference acceptability tolerance. By applying the Measurement Uncertainty Correction, the user essentially loosens the gamma comparison criteria from 2%/2 mm to 3%/2 mm, which is the recommendation from TG‐218. The failed measurements are those that produce global %GPs <95% under the same conditions. Two hundred and sixty two VMAT beams were analyzed, including 230 passed and 32 failed beams. The work was repeated for 167 HT plans, including 142 passed and 25 failed plans (see appendix).

**Table 1:** Overview of PSQA measurements assessed.

Modality	Accelerator	Beams passed	Beams failed	MLC	TPS	Algorithm
HT	Hi‐Art 2	142	25	Binary MLC	Tomotherapy	CCC
VMAT	Varian iX	230	32	Millenium 120	Eclipse v13.5	AAA

Abbreviations: HT, helical tomotherapy; MLC, multileaf collimator; PSQA, patient‐specific pretreatment quality assurance; TPS, treatment planning system; VMAT, volumetric modulated arc therapy.

%GPs were calculated using multiple criteria (1%/1 mm, 2%/2 mm, 2%/3 mm, 3%/2 mm, 3%/3 mm, and 5%/3 mm), multiple LDTs (5% and 10%) and with ∆D both determined globally (i.e. using maximum dose value in distribution) and locally. MADD*_b_* and MADD*_γ_* determined agreement indices were calculated using the same criteria pairs as were used for the global and local gamma evaluations. These criteria were selected based on local and survey‐reported practices.^2^, ^4^, ^23^

D&C agreement indices were calculated using the same criteria pairs as were used for the gamma and MADD evaluations, with ∆D criteria replaced as summarized in Table 2. The dose criteria for each dose region was selected for approximate equality in terms of dose in “absolute” dose. They were chosen such that the local dose difference corresponded to approximately the same global dose difference; that is a 7% dose difference in the HG region (centered around the 70% isodose) is about equal to a 5% difference in maximum dose (0.7 × 0.07 = 0.049). This is why, for example, 5%, 7%, 10%, and 15% were used for HD, HG, MD, and LD in one case.

**Table 2:** Dose difference criteria used for D&C evaluation.

∆Dγ (%)	∆D_HD_ (%)	∆D_HG_ (%)	∆D_M_ (%)_D_	∆D_LD_ (%)
1	1	1.5	3	6
2	2	3	5	8
3	3	4	5	10
5	5	7	10	15

Abbreviation: D&C, divide and conquer.

The relationships between agreement indices calculated using varying LDT for local and global gamma analysis, calculated using varying criteria for each dose comparison technique, and calculated using varying dose comparison techniques for each criteria were quantified using ordinary least squares regression. Correlation (or similarity in behavior, in terms of identifying results as demonstrating high or low agreement) was assessed using coefficients of determination R^2^ (representing the variation in the dependent variable that can be explained by variation in the independent variable) and *P*‐values. An R^2^ ≥ 0.64 was selected as a correlation threshold, consistent with the adoption of a Pearson's correlation coefficient of 0.8 as a minimum clinically acceptable value in the literature.^11^, ^25^ A significance level α = 0.05 was selected as the threshold for significance, with Ŝidák corrections applied to account for multiple comparisons.

When evaluating relationships between comparison techniques and agreement criteria, global and local %GPs were calculated using 10% LDT, the most frequently reported threshold value in the literature.^2^, ^14^, ^23^, ^25^

## 3 RESULTS

This section only included the results from the VMAT plans. The HT results can be found in the appendix. Table 3 shows the mean agreement indices that resulted from evaluating 262 VMAT PSQA measurements using the various comparison methods investigated in this study. Table 4 summarized correlation between 5% and 10% LDT‐calculated %GPs for VMAT plans (P* ≤ *α in all cases). Calculated R^2^ values and Ŝidák‐corrected significance results for relationships between dose comparison techniques for 2%/2 mm are presented in Table 5. Calculated R^2^ values and Ŝidák‐corrected significance results for relationships between agreement indices calculated for varying criteria for each dose comparison technique are presented as appendix.

**Table 3:** Mean agreement indices (%) and standard deviation of 262 VMAT arcs for each dose comparison technique.

Index	1%/1 mm	2%/2 mm	2%/3 mm	3%/2 mm	3%/3 mm	5%/3 mm
γ_global*,*LDT = 5%_	58.3* ± *9.2	94.8* ± *2.9	97.3* ± *1.8	98.1* ± *2.0	99.1* ± *1.1	99.8* ± *0.4
γ_global*,*LDT = 10%_	53.0* ± *9.5	93.3* ± *4.1	96.6* ± *2.5	97.5* ± *2.9	98.7* ± *1.6	99.8* ± *0.6
γ_local*,*LDT = 5%_	17.1* ± *6.1	60.4* ± *9.0	73.3* ± *8.2	69.6* ± *9.3	79.8* ± *8.1	87.4* ± *7.6
γ_local*,*LDT = 10%_	20.1* ± *6.4	69.1* ± *7.5	81.5* ± *5.2	78.9* ± *7.0	87.8* ± *4.5	94.4* ± *3.5
D&C	58.5* ± *13.1	92.4* ± *7.6	95.5* ± *5.1	96.4* ± *5.6	97.9* ± *3.9	99.4* ± *1.9
MADD_b_	86.9* ± *9.8	99.5* ± *0.6	99.8* ± *0.3	99.9* ± *0.2	100* ± *0.1	100* ± *0.03
MADD*_γ_*	75.8* ± *14.2	97.7* ± *1.6	98.9* ± *1.0	99.2* ± *0.7	99.3* ± *6.1	100* ± *0.1

Abbreviations: D&C, divide and conquer; LDT, lower dose thresholds; MADD, maximum allowed dose difference; VMAT, volumetric modulated arc therapy.

**Table 4:** R^2^ for relationship between %GPs using 5% LDT and 10% LDT for 262 VMAT arcs.

Index	1%/1 mm	2%/2 mm	2%/3 mm	3%/2 mm	3%/3 mm	5%/3 mm
γ_global_	0.702	0.947	0.955	0.980	0.983	0.985
γ_local_	0.865	0.481	0.287	0.393	0.212	0.112

Abbreviations: LDT, lower dose thresholds; VMAT, volumetric modulated arc therapy.

**Table 5:** R^2^ for relationship between agreement indices for varying dose comparison techniques using 2%/2 mm for 262 VMAT plans.

	*γ*_local_	D&C	MADD_b_	MADD*_γ_*
*γ*_global_	0.652	0.538	0.133	0.009
*γ*_local_		0.404	0.043	0.000
D&C			0.043	0.001
MADD_b_				0.626

Abbreviations: D&C, divide and conquer; MADD, maximum allowed dose difference; VMAT, volumetric modulated arc therapy.

The following results were observed:

- The mean agreement indices calculated at 5%/3 mm were nearly 100% for every case except for local gamma analysis.
- Applying more strict gamma criteria results in higher standard deviation of data.
- Global gamma evaluation technique with various gamma criteria behave similarly regardless of LDT.
- The correlation between 5% and 10% LDT in local gamma calculations decreased with increasing ∆D and DTA, suggesting that large percentage dose differences were more prevalent in low dose regions. Local gamma analysis had the least variation in behavior with criteria selection.
- Correlation of 1%/1 mm or 5%/3 mm with other criteria was generally poor for all techniques except local gamma evaluation, where VMAT shows lower correlation than HT (see appendix) at 1%/1 mm. Correlation at 2%/2mm was generally poor between gamma, D&C and MADD dose comparison techniques in VMAT plans.
- Agreement indices calculated for 2%/2 mm and 2%/3 mm, and 3%/2 mm and 3%/3 mm were correlated for most comparison techniques.

## 4 DISCUSSION

The mean %GPs calculated for multiple criteria in this study were consistent with values reported in the literature for VMAT technique.^23^ The differences between the global %GP with 5% LDT were consistent with results reported by Crowe et al.^23^ using ArcCheck measurements, particularly at 1%/1 mm.

The behavior of %GP with varying LDT is consistent with results described by Son et al.,^26^ where increasing the LDT resulted in an increase of agreement indices for global gamma evaluation, and a decrease in agreement indices for local gamma evaluation. Choice of LDT may potentially have large a impact on calculated %GP, particularly with stricter agreement criteria, for example, tighter tolerances or local (rather than global) gamma calculation.

The coefficient of determination values in Tables 4 and 5 and the appendix identify whether an agreement index calculated using one technique, criteria pairs or LDTs is predictive of the agreement index calculated using a different technique, criteria pair or LDT; or in other words, whether there is consistency in which plans have relatively high or low agreement indices, regardless of the technique, criteria or LDT selection^23^. A low coefficient of determination (R^2^ < 0.64), i.e. poor correlation, suggests that the two dose comparison techniques, criteria pair or LDT exhibit different behaviors, and thus may exhibit different sensitivity to dose errors.

Assuming dose delivery accuracy (or “plan deliverability”) is intrinsic to a treatment plan, it would be reasonable to assume correlation of agreement indices across different criteria. The correlation between agreement indices calculated using local gamma evaluation supports this assumption, and may assist in explaining, for example, why machine learning has been successfully used to predict local gamma evaluation agreement indices from treatment parameters.^27^

The difference in behavior of local and global gamma evaluation techniques identified here is consistent with observations in the literature,^11^, ^12^ which report that the two techniques exhibit varying specificity and sensitivity to dose delivery errors. Stasi et al.^11^ reported improved sensitivity to errors when using local gamma evaluation, compared to global gamma evaluation. Nelms et al.^12^, ^16^ identified the use of global normalization as a contributor to insensitivity to dose errors in anatomic regions‐of‐interest.

**Figure 1:**
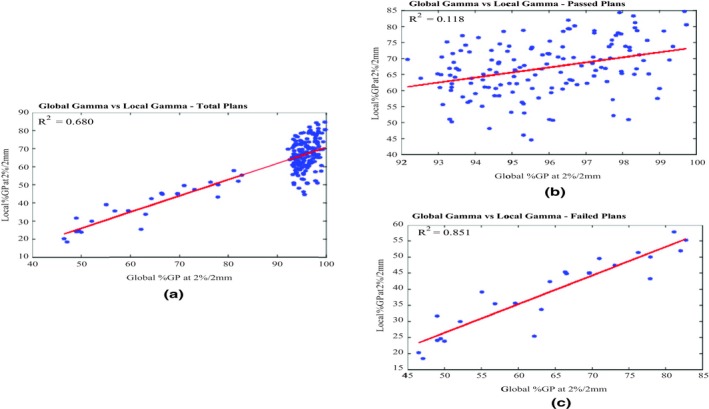
(a) Global versus local %GPs at 2%, 2 mm for all volumetric modulated arc therapy (VMAT) plans, (b) global versus local %GPs at 2%, 2 mm for passed VMAT plans and (c) global versus local %GPs at 2%, 2 mm for failed VMAT plans.

Among the 262 VMAT PSQA measurements analyzed, 32 measurements were included which failed the initial global gamma evaluation using 2%, 2 mm and LDT 5%. The failed plans were selected from our most recent QA records, and their number approximates the same clinical occurrence ratio of failed plans among all PSQA in our clinic. With the inclusion of the failed plans, the overall behavior of the performed analyses is consistent with those with passed plans only. However in Table 5, the correlation may be overestimated due to the much lower pass rates and the low proportion of the failed plans compared with the passed ones, as shown in Fig. 1. The R^2^ values become less comparable when the sample size varies, therefore cannot be solely replied upon. According to the figure, it also indicated that gamma pass rates of 90% for global gamma falls to an average of approximately 60% when local gamma metric is used.

The search for better dose comparison metrics has been ongoing for years. TG‐218^5^ also cited several other tools proposed after the gamma test, including the gradient compensation method,^28^ the normalized agreement test (NAT)^29^ and the Bakai method.^30^ They all pointed out the weaknesses of the gamma test and hence the desire for an improved dose comparison matrix. To a radiation oncology treatment center currently employing the common 3%/3 mm global gamma evaluation technique with 10% LDT,^2^, ^3^, ^4^ the results of this study demonstrated:

- The expected change in agreement indices for the global gamma evaluation can be estimated if the center adopts a tighter (2%/2 mm) criteria, as recommended by Nelms et al.^12^ (98.7* ± *1.6 to 93.3* ± *4.1).
- The D&C technique or the local gamma evaluation may exhibit increased sensitivity to dose errors^11^, ^12^ and thus may be preferable to identify undesirable plans. According to Table 3, a 1%‐10% decrease in pass rate may be expected for the criteria of 3%, 3 mm based on the data presented in this work.

## 5 CONCLUSIONS

This work has evaluated alternative dose comparison techniques and compared with the standard gamma analysis technique. It was found that each individual dose comparison technique behaves differently from each other. The local gamma analysis had the strongest correlation between results calculated using different acceptance criteria amongst the dose comparison techniques. Radiation oncology treatment centers looking to compare between different dose comparison techniques, criteria or LDT may use the results of this study to estimate the expected differences in calculated agreement indices and possible variations in behavior to delivery dose errors.

## CONFLICT OF INTEREST

The authors have no relevant conflicts of interest to disclose.

## Figures and Tables

**Figure A1:**
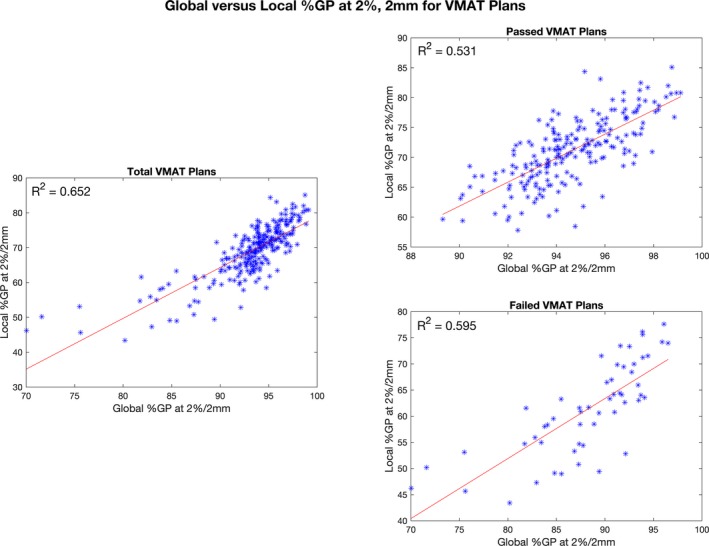
(a) Global vs. local %GPs at 2%, 2 mm for all HT plans, (b) Global versus local %GPs at 2%, 2 mm for passed HT plans. and (c) Global versus local %GPs at 2%, 2 mm for failed HT plans.

## APPENDIX 1

### 1.1

Table A1 shows the mean agreement indices that resulted from evaluating 167 HT PSQA measurements using the various comparison methods investigated in this study. There are more spread in the data of HT plans than VMAT ones for all techniques and criteria. For tighter criteria, there are more spread in the data for both treatment modalities.

**Table:** Table A1 Mean agreement indices (%) and standard deviation of 167 HT plans for each dose comparison technique

Index	1%/1 mm	2%/2 mm	2%/3 mm	3%/2 mm	3%/3 mm	5%/3 mm
*γ*_global*,*LDT=5%_	51.0 *±* 16.6	91.7 *±* 11.8	95.1 *±* 8.7	97.3 *±* 5.5	98.5 *±* 3.6	99.8 *±* 0.7
*γ*_global*,*LDT=10%_	50.8 *±* 15.9	91.1 *±* 12.4	94.9 *±* 8.9	96.9 *±* 6.6	98.3 *±* 4.2	99.8 *±* 0.9
*γ*_local*,*LDT=5%_	18.8 *±* 7.2	57.8 *±* 14	71.6 *±* 12.8	67.3 *±* 14.8	78.1 *±* 12.9	85.9 *±* 11.6
*γ*_local*,*LDT=10%_	20.5 *±* 7.2	62.7 *±* 13.5	76.8 *±* 11.8	72.9 *±* 13.9	83.4 *±* 11.5	91.1 *±* 9.5
D&C	57.1 *±* 15.0	90.2 *±* 11.0	94.2 *±* 8.2	95.7 *±* 7.1	97.6 *±* 5.2	99.5 *±* 1.9
MADD_b_	76.8 *±* 16.7	97.6 *±* 5.3	98.6 *±* 3.3	99.0 *±* 7.1	99.7 *±* 0.9	100 *±* 0.1
MADD*γ*	62.8 *±* 16.6	93.0 *±* 10.3	95.5 *±* 8.1	97.8 *±* 4.7	98.7 *±* 3.49	9.9 *±* 0.5

Table A2 summarised correlation between 5% and 10% LDT‐calculated gamma agreement indices for HT plans (*p ≤ α* in all cases). For global gamma evaluation, the results of 5% or 10% LDT calculations are correlated for all criteria, i.e. the techniques behave similarly regardless of LDT. For local gamma evaluation, the correlation between 5% and 10% LDT calculations decreased with increasing ∆D and DTA, suggesting that large percentage dose differences were more prevalent in low dose regions. The behaviour variation between HT and VMAT cohorts possibly suggests a difference in low dose calculation or delivery accuracy between the two treatment modalities. (Tables A3 and A4)

Correlation was poor for most relationships in VMAT. Better overall correlation was observed for HT plans, with MADD_b_ vs. MADD_*γ*_ being the highest (Table A5; Fig A1).

**Table:** Table A2 R^2^ for relationship between %GP using 5% LDT and 10% LDT for 167 HT plans

Index	1%/1 mm	2%/2 mm	2%/3 mm	3%/2 mm	3%/3 mm	5%/3 mm
*γ*global	0.982	0.981	0.983	0.975	0.982	0.987
*γ*local	0.973	0.926	0.884	0.920	0.878	0.845

**Table:** Table A3 R^2^ for relationship between agreement indices for varying dose comparison techniques using 2%/2 mm for HT (lower left, shaded) and VMAT (upper right)

	*γ*global	*γ*local	D&C	MADDb	MADD*γ*
*γ*global		0.652	0.538	0.133	0.009
*γ*local	0.680	0.404		0.043	0
D&C	0.868	0.652		0.043	0.001
MADD_b_	0.844	0.567	0.717		0.626
MADD*γ*	0.874	0.627	0.751	0.943	

Notes

Sˆid′ak corrected significance level *α*_*sid*_ **= 0.003, p_sid_ *α*_sid_ in all cases except for HT where MADD_b_: p_sid_ (2%/2 mm vs. 3%/2 mm) = 0.039, MADD_b_: p_sid_(2%/3 mm vs. 3%/2 mm) = 0.586, MADD_b_: p_sid_ (3%/2 mm vs. 3%/3 mm) = 0.152, MADD_b_: p_sid_ (3%/2 mm vs. 5%/3 mm) = 0.062 and VMAT where MADD_*γ*_ : p_sid_ (2%/3 mm vs. 3%/3 mm) = 0.326, MADD_*γ*_ : p_sid_ (3%/2 mm vs. 3%/3 mm) = 0.863, MADD_*γ*_ : p_sid_ (3%/3 mm vs. 5%/3 mm) = 0.087.

**Table:** Table A4 R^2^ for relationship between %GP/BAI using one pair of particular criteria and other pairs of criteria. Tomotherapy results in lower left, VMAT results in upper right

Colour	HT	VMAT				
Gglobal	1%/1 mm	2%/ 2 mm	2%/3 mm	3%/2 mm	3%/3 mm	5%/3 mm
1%/1 mm	0.628	0.474	0.500	0.407	0.305	0.474
2%/2 mm	0.760		0.860	0.897	0.769	0.567
2%/3 mm	0.682	0.967		0.812	0.840	0.606
3%/2 mm	0.612	0.903	0.852		0.863	0.649
3%/3 mm	0.558	0.873	0.887	0.947		0.649
5%/3 mm	0.235	0.349	0.306	0.531	0.526	
Glocal	1%/1 mm	2%/ 2 mm	2%/3 mm	3%/2 mm	3%/3 mm	5%/3 mm
1%/1 mm		0.440	0.541	0.735	0.546	0.546
2%/2 mm	0.841		0.853	0.952	0.808	0.720
2%/3 mm	0.713	0.954		0.874	0.922	0.784
3%/2 mm	0.800	0.979	0.948		0.867	0.814
3%/3 mm	0.680	0.928	0.977	0.957		0.872
5%/3 mm	0.575	0.822	0.874	0.890	0.942	
D&C	[1%HD…]/1 mm	[2%HD…]/2 mm	[2%HD…]/3 mm	[3%HD…]/2 mm	[3%HD…]/3 mm	[5%HD…]/3 mm
[1%HD…]/1 mm		0.533	0.410	0.456	0.328	0.172
[2%HD…]/2 mm	0.763		0.722	0.753	0.562	0.256
[2%HD…]/3 mm	0.685	0.936		0.775	0.781	0.363
[3%HD…]/2 mm	0.700	0.915	0.903		0.753	0.324
[3%HD…]/3 mm	0.617	0.802	0.885	0.899		0.437
[5%HD…]/3 mm	0.294	0.397	0.475	0.537	0.667	
MADD_b_	1%/1 mm	2%/ 2 mm	2%/3 mm	3%/2 mm	3%/3 mm	5%/3 mm
1%/1 mm		0.090	0.056	0.091	0.054	0.184
2%/2 mm	0.746		0.861	0.695	0.693	0.112
2%/3 mm	0.712	0.993		0.713	0.793	0.116
3%/2 mm	0.012	0.028	0.029		0.817	0.198
3%/3 mm	0.464	0.784	0.081	0.037		0.214
5%/3 mm	0.189	0.279	0.292	0.018	0.529	
MADD*γ*	1%/1 mm	2%/ 2 mm	2%/3 mm	3%/2 mm	3%/3 mm	5%/3 mm
1%/1 mm		0.197	0.128	0.094	0.118	0.034
2%/2 mm	0.774		0.899	0.611	0.002	0.123
2%/3 mm	0.719	0.987		0.610	0.002	0.196
3%/2 mm	0.613	0.888	0.887		0.002	0.405
3%/3 mm	0.549	0.854	0.876	0.979		0.001
5%/3 mm	0.246	0.347	0.376	0.480	0.506	

Sˆid′ak corrected significance level *α*_*sid*_ = 0.005, p_sid_ *α*_sid_ in all cases except for HT where 2%/2 mm: p_sid_ (D&C vs. G_global_) = 0.014, 2%/3 mm: p_sid_ (D&C vs. G_global_) = 0.008, 3%/2 mm: p_sid_ (MADD_*γ*_ vs. MADDb) = 0.061, 5%/3 20 mm: p_sid_ (D&C vs. G_global_) = 0.022, 5%/3 mm: p_sid_ (MADD_*γ*_ vs. G_global_) = 0.039, 5%/3 mm: p_sid_ (MADD_*γ*_ vs. D&C) = 0.008 and for VMAT where 3%/3 mm: p_sid_ (MADD_*γ*_ vs. G_global_) = 0.152, 3%/3 mm: p_sid_ (MADD_*γ*_ vs. MADDb) = 0.090.

**Table:** Table A5 R^2^ for relationship between %GP/BAI of one particular dose comparison technique and other dose comparison techniques. Tomotherapy results in lower left, VMAT results in upper right

	HT	VMAT			
1%/1 mm	G_global_	G_local_	D&C	MADD_b_	MADD_*γ*_
G_globa_		0.488	0.585	0.124	0.037
Glocal	0.684		0.642	0.002	0.043
D&C[1%HD…]/1 mm	0.844	0.657		0.005	0.014
MADD_b_	0.808	0.537	0.765		0.796
MADD*γ*	0.813	0.534	0.721	0.959	
2%/2 mm	Gglobal	Glocal	D&C	MADD_b_	MADD*γ*
Gglobal		0.652	0.538	0.133	0.009
Glocal	0.680		0.404	0.043	0
D&C[2%HD…]/2 mm	0.868	0.652		0.043	0.001
MADD_b_	0.844	0.567	0.717		0.626
MADD*γ*	0.874	0.627	0.751	0.943	
2%/3 mm	Gglobal	Glocal	D&C	MADD_b_	MADD*γ*
Gglobal		0.642	0.465	0.168	0.060
Glocal	0.702		0.306	0.049	0.005
D&C[2%HD…]/3 mm	0.866	0.635		0.029	0
MADD_b_	0.879	0.592	0.756		0.637
MADD*γ*	0.918	0.657	0.786	0.955	
3%/2 mm	Gglobal	Glocal	D&C	MADD_b_	MADD*γ*
Gglobal		0.602	0.629	0.300	0.135
Glocal	0.613		0.390	0.127	0.058
D&C[3%HD…]/2 mm	0.830	0.622		0.124	0.038
MADD_b_	0.025	0.012	0.019		0.661
MADD*γ*	0.824	0.610	0.713	0.029	
3%/3 mm	Gglobal	Glocal	D&C	MADD_b_	MADD*γ*
Gglobal		0.500	0.473	0.202	0
Glocal	0.611		0.261	0.104	0
D&C[3%HD…]/3 mm	0.773	0.574		0.041	0
MADD_b_	0.812	0.457	0.586		0.002
MADD*γ*	0.875	0.609	0.641	0.854	
5%/3 mm	Gglobal	Glocal	D&C	MADD_b_	MADD*γ*
Gglobal		0.516	0.288	0.122	0.137
Glocal	0.224		0.216	0.088	0.131
D&C[5%HD…]/3 mm	0.395	0.253		0.191	0.078
MADD_b_	0.784	0.251	0.374		0.396
MADD*γ*	0.841	0.362	0.324	0.829	
